# Gentiopicroside alleviates type 2 diabetes mellitus by attenuating oxidative stress and reshaping gut microbiota in high-fat diet and streptozotocin-induced mice

**DOI:** 10.3389/fnut.2026.1768857

**Published:** 2026-02-26

**Authors:** Xing Wang, Dongmei Long, Xingcan Peng, Linlin Wu, Qitong Xie, Siyao Luo, Huijuan Li, Maoting Zhou, Tian Zhou

**Affiliations:** 1 Department of Pharmacology, School of Pharmacy, North Sichuan Medical College, Nanchong, China; 2 Nanchong Key Laboratory of Disease Prevention, Control and Detection in Livestock and Poultry, Nanchong Vocational and Technical College, Nanchong, China; 3 The Affiliated Children’s Hospital of Zhengzhou University, Zhengzhou, China

**Keywords:** gentiopicroside, gut microbiota, Nrf2/Keap1 signaling, oxidative stress, type 2 diabetes mellitus

## Abstract

**Introduction:**

The rising incidence of T2DM, along with the limited efficacy and side effects of current drugs, demands new therapies. Gentiopicroside (GPS) has been shown to improve T2DM and its chronic complications; however, whether these effects are related to modulation of the gut microbiota (GM) remains unclear. Herein, the relationship between the therapeutic effects of GPS on T2DM and GM alterations was investigated using a C57BL/6J mouse model of T2DM induced by a high-fat diet combined with streptozotocin (STZ).

**Methods:**

A T2DM model was induced in C57BL/6J mice by high-fat diet combined with STZ. Biochemical methods were used to determine glucose metabolism and oxidative stress-related indices in serum and liver; Western blot was employed to detect the expression of proteins related to the Nrf2/Keap1 signaling pathway in the liver; 16S rRNA high-throughput sequencing was used to detect and analyze gut microbiota in mouse feces.

**Results:**

The results demonstrated that 8 weeks of GPS supplementation significantly reduced blood glucose and insulin levels, improved glucose tolerance and insulin resistance, alleviated liver pathology, enhanced the activity of antioxidant enzymes in serum and liver, increased antioxidant substance levels, and decreased MDA content. Moreover, GPS markedly upregulated the expression protein of Nrf2, HO-1, and NQO1, while downregulating the Keap1 expression in the liver. High-throughput 16S rRNA sequencing further revealed that GPS significantly increased the Chao1 index and Observed_otus index, showed a trend of improving indices such as Shannon, Simpson, Pielou-e, Goods-coverage, and improved *β*-diversity in fecal samples from T2DM mice. GPS increased the Firmicutes-to-Bacteroidetes (F/B) ratio and reduced the relative abundance of Verrucomicrobiota, Cyanobacteria and Unclassified at the phylum level. At the genus level, GPS increased the relative abundance of Lactobacillus, HT002, Dubosiella, and reduced that of Muribaculaceae_unclassified, Akkermansia, Desulfovibrio, Muribaculum Alloprevotella. Correlation analysis further indicated that the anti-T2DM effects of GPS were closely related to improvements in GM diversity and composition.

**Conclusion:**

In conclusion, these results indicated that GPS can reshape the structural composition and diversity of GM, activate the hepatic Nrf2/Keap1 pathway, and maintain glucose homeostasis.

## 1 Introduction

Type 2 diabetes mellitus (T2DM), in particular, is part of diabetes mellitus that has turned into a major public health concern on a global scale. Due to unhealthy diets and sedentary lifestyles, its prevalence has steadily increased in recent decades. T2DM is a complex chronic disease involving multiple pathogenic factors, and is primarily defined by persistent hyperglycemia, compromised pancreatic *β*-cell function, relative or absolute insulin insufficiency, as well as insulin resistance. These factors can ultimately lead to damage in multiple organs and progress to serious complications such as neuropathy, nephropathy, and retinopathy (1). According to the Global Diabetes Map released by the International Diabetes Federation in 2025, the number of diabetic patients worldwide was 643 million in 2024, and this number is projected to rise to 853 million by 2050, equivalent to one-eighth of the global population (2). Currently, biguanides, *α*-glucosidase inhibitors, sulfonylureas, and insulin analogues are commonly used in clinical practice to control blood glucose levels and slow disease progression. However, these drugs only temporarily alleviate hyperglycemia symptoms. Long-term use may further reduce insulin sensitivity and worsen insulin resistance. It can also cause adverse effects such as vomiting, hypoglycemia, gastrointestinal discomfort, fractures, and heart failure (1, 3). Accordingly, there is an urgent need to explore safe and effective new drugs and intervention strategies for preventing and controlling T2DM, with the goal of reducing the risk of disease progression and associated complications.

An increasing body of evidence clearly shows that oxidative stress plays a fundamental role in the pathological process of T2DM (4). An appropriate level of free radicals is crucial for maintaining the redox balance of the body. However, under persistent hyperglycemia, excessive amounts of free radicals are generated. When their levels exceed the body’s antioxidant capacity, they promote the progression of T2DM. The liver, as one of the three primary target organs of insulin, plays an important role in regulating glucose homeostasis. Excessive reactive oxygen species (ROS) induced by chronic hyperglycemia can cause hepatocyte injury and trigger inflammatory and fibrotic responses, thereby contributing to liver fibrosis (5). In addition, ROS can interact with biological macromolecules in the liver, disrupt enzymatic activity, impair membrane function, induce lipid peroxidation, and cause liver damage of varying severity, all of which accelerate the progression of T2DM (6). Meanwhile, studies have shown that treatment with antioxidants in diabetic mice significantly improves diabetic symptoms, increases insulin levels, and alleviates hepatic steatosis (7). The gut microbiota (GM), the largest and most complex microecosystem in the human gastrointestinal tract, plays a key role in maintaining intestinal function and energy metabolism (8). An imbalance in GM composition is strongly correlated with the onset, progression, and treatment of T2DM (8, 9). Persistent hyperglycemia can lead to gut dysbiosis, increasing intestinal permeability, disrupting the integrity of the intestinal barrier, triggering intestinal inflammation, and altering the synthesis of various bioactive metabolites (10, 11). These changes can negatively affect energy metabolism and insulin sensitivity, thereby promoting the occurrence and development of T2DM. Therefore, oxidative stress and GM have become key areas of focus in the search for new drugs to prevent and treat T2DM. Identifying effective strategies to regulate both aspects in a beneficial and controlled manner remains an urgent area of ongoing research.

Natural products extracted from traditional Chinese medicines offer unique advantages and significant potential in the development of new drugs and the treatment of chronic diseases due to their low toxicity and minimal side effects. A natural iridoid glycoside compound known as gentiopicroside (GPS) is obtained from Gentiana manshurica Kitagawa, which is a traditional Chinese medicinal herb commonly employed in China. Animal experiments have confirmed that GPS has multiple pharmacological activities, including antifungal, antibacterial, anti-inflammatory, hepatoprotective, and free radical scavenging effects (12, 13). Notably, numerous studies have also reported the therapeutic potential of GPS in alleviating diabetic symptoms. For example, Xu et al. demonstrated that GPS improves glucose and lipid metabolism in T2DM by targeting fibroblast growth factor receptor 1 (14). GPS also targets progestin and adipoQ receptor 3 to activate the phosphatidylinositol 3-kinase (PI3K)/protein kinase B (AKT) signaling pathway, thereby ameliorating dysregulated glucose and lipid metabolism in diabetic mice induced by STZ and a HFD (15). Our previous studies have shown that GPS improves diabetic symptoms in T2DM mice by inhibiting gluconeogenesis through regulation of the PI3K/AKT/forkhead Box O1 (FOXO1) signaling pathway (16). GPS significantly alleviates diabetic peripheral neuropathy, diabetic nephropathy, diabetic retinopathy, and non-alcoholic fatty liver disease, and promotes the healing of diabetic wounds, these effects are linked to the compound’s capacity to inhibit inflammatory responses and oxidative stress, as well as regulate the PPARα/HIF1 signaling pathway and the AKT1/HIF-1α/VEGF signaling pathway (17–19). In addition, GPS could inhibit the inflammatory response and fibrosis of cardiac cells by targeting Smad3 phosphorylation, thereby improving cardiac function (20); GPS exerted a certain therapeutic effect on adjuvant-induced arthritis by inhibiting inflammation and oxidative stress in rats (21). GPS alleviated chronic 3,5-diethoxycarbonyl-1,4dihydrocollidine diet-induced bile duct disease by improving ductal reactivity, reducing periductal fibrosis, and mitigating oxidative stress and inflammatory response (22). GPS improved high-fat diet-induced non-alcoholic fatty liver disease in mice by regulating the serum metabolome and the composition of gut microbiota (23). GPS alleviated dextran sulfate sodium-induced ulcerative colitis and secondary liver injury in mice by enhancing the intestinal barrier and regulating gut microbiota (24). Furthermore, GPS exerts its pharmacological effects by regulating the Nrf2 signaling pathway (25, 26); however, no studies have yet revealed the therapeutic mechanism of GPS in T2DM from the dual perspective of Nrf2/Keap1 pathway activation and overall GM structure modulation. Therefore, we evaluated the effects of GPS on the composition of intestinal microflora and the Nrf2/Keap1 signaling pathway in STZ- and HFD-induced diabetic mice, and aimed to provide evidence for further clarifying the mechanism of GPS in improving T2DM.

## 2 Materials and methods

### 2.1 Reagents and antibodies

GPS (C16H20O9, MW: 356.3, purity:≧98%) and STZ (purity≧99%) were purchased from Yuanye Bio-Technology Co. Ltd. (Shanghai, China). The high-fat feed containing 40.7% normal feed, 27% lard, 13.5% casein, 10.8% full-fat milk powder and 8% sucrose was purchased from Shanghai Shuyu Biotechnology Co., Ltd. (Shanghai, China). The nucleoprotein extraction kit was obtained from Beijing Biosynthesis Biotechnology Co., Ltd. (Nanjing, China). The insulin content determination kit was obtained from Camilo Biological (Nanjing, China). The total antioxidant capacity (T-AOC), superoxide dismutase (SOD), MDA, glutathione (GSH), aspartate aminotransferase (AST) and alanine aminotransferase (ALT) detection kit were acquired from Nanjing Jiancheng Bioengineering Institute (Nanjing, Jiangsu, China). Antibodies against nuclear factor E2 associated factor 2 (Nrf2) and heme oxygenase-1 (HO-1) were provided by Abcam (Cambridge, Cambridgeshire, UK). Antibodies to Kelch like epichlorohydrin associated protein-1 (Keap1) and NADPH quinone oxidoreductase 1 (NQO1) were purchased from Cell Signaling Technology (Danvers, MA, USA). The remaining reagents and kits for western blot were purchased from Applygen Technologies (Beijing, China).

### 2.2 Animals

Thirty male C57BL/6J mice, aged 7–8 weeks and weighing 24 ± 2 g, were acquired from Henan Skobes Biotechnology Co., Ltd. (Henan, China, production license: SCXK (Yu) 2020-0005). The mice were kept in a standard animal facility at North Sichuan Medical College under controlled conditions (humidity: 55 ± 10%, temperature: 23 ± 2 °C, and a 12-h light/dark cycle). All experimental protocols were strictly performed in line with the Guidelines for the Care and Use of Laboratory Animals (GB14925-2001 and MOST 2006a), and the animal experiments were approved by the Animal Ethics Committee of North Sichuan Medical College (Approval No. NSMC2024132).

### 2.3 Experimental design

A classic method involving a HFD combined with STZ was used to induce a T2DM model in C57BL/6J mice. The specific procedure was consistent with previously described methods (Wang et al., 2023). Once the model was successfully established, the diabetic mice were randomly categorized into two groups: the T2DM model group (Mod) and the GPS treatment group (50 mg/kg), which received GPS via intragastric administration. The dosage of GPS was determined based on previous studies (14, 16). The normal control (Nor) group consisted of C57BL/6J mice fed a normal diet and administered citrate buffer via intraperitoneal injection. GPS was dissolved in 0.5% carboxymethylcellulose sodium (CMC-Na) and administered by gavage for 8 weeks. Both the Nor and Mod groups received an equivalent volume of 0.5% CMC-Na. Throughout the experiment, body weight, fasting blood glucose (FBG), and postprandial random blood glucose (RBG) were measured weekly for all groups. After 8 weeks, the mice were anesthetized via intraperitoneal injection of urethane at a dose of 1 g/kg, subsequent to respiratory arrest, death was verified via cervical dislocation, in compliance with the American Veterinary Medical Association (AVMA) Guidelines. After the mice were euthanized, they were weighed, followed by blood collection via orbital sinus puncture. The livers were excised and weighed. Each liver was divided such that part was fixed in 4% neutral paraformaldehyde, and the remaining tissue was stored at −80 °C for biochemical analysis as well as protein detection. Calculation of the liver index was performed utilizing the formula shown below: liver index = liver weight (mg)/body weight (g).

### 2.4 Oral glucose tolerance test (OGTT) and insulin tolerance test (ITT)

The OGTT was conducted during the 7th week of drug administration. After fasting the mice for 8 h, FBG was measured as the 0-min blood glucose value. A glucose solution (2 g/kg) was then administered via oral gavage. Blood samples were collected at 30, 60, 90, and 120 min after glucose administration, and the blood glucose concentration was determined using the glucose oxidase method. A glucose tolerance curve was plotted, and the area under the curve (AUC) was calculated. The ITT was conducted during the 8th week of drug administration. After fasting the mice for 6 h, FBG was measured as the 0-min blood glucose value. This was followed by a subcutaneous injection of insulin (0.75 IU/kg). The subsequent procedures were consistent with those used in the OGTT.

### 2.5 Biochemical analysis in serum

After the whole blood was left to stand at room temperature for 2 h, it was centrifuged at 5000 rpm at 4 °C for 10 min to separate the supernatant. Subsequently, the serum levels of GSH, MDA, and insulin in the serum, as well as the activities of T-AOC and SOD were measured strictly according to the kit instructions.

### 2.6 Determination of oxidative stress index in liver

A total of 100 mg of liver tissue from each mouse was weighed into a 1.5 mL Eppendorf (EP) tube, and 900 μL of pre-cooled physiological saline along with steel beads was added. The mixture was thoroughly homogenized using a mechanical homogenizer and then allowed to stand on ice for 40 min. It was subsequently centrifuged at 12,000 rpm for 10 min at 4 °C to obtain the supernatant. The contents of GSH, T-AOC, SOD, and MDA in the liver were determined strictly according to the instructions of the assay kits. Next, 10 μL of the supernatant was diluted tenfold, and the protein concentration was measured using the bicinchoninic acid (BCA) method. The final results of oxidative stress-related indicators in the liver were normalized to the protein concentration.

### 2.7 Histological analysis

The liver tissues were fixed in 4% paraformaldehyde for 24 h, embedded in paraffin, and sectioned into 4-μm-thick slices using a microtome. Subsequently, hematoxylin and eosin (HE) staining and periodic acid-Schiff (PAS) staining were performed on the liver sections strictly following the kit instructions. Finally, pathological changes in the liver were evaluated by observation and photography under a microscope (Nikon ECLIPSE E100, Nikon, Japan).

### 2.8 Nuclear and cytoplasmic proteins extraction assay

Nuclear and cytoplasmic proteins were extracted from cultured cells or fresh tissue samples using a commercial nuclear extraction kit (Bioss, C5024). Livers from 6 randomly selected mice in each group were accurately weighed (50 mg) and placed into clean 1.5 mL EP tubes. After adding 500 μL of PBS, the mixture was homogenized with a mechanical homogenizer at 4 °C, incubated on ice for 10 min, and then centrifuged at 1000 r/min for 3 min to collect cells. Next, 200 μL of cytoplasmic protein extraction reagent was added, and the pellet was fully resuspended by vigorous vortexing, followed by incubation on ice for 10 min. After vortexing at high speed for 10 s, centrifugation was performed at 12,000 r/min at 4 °C for 10 min. The collected supernatant was cytoplasmic protein, which was stored at −70 °C for subsequent analysis. Subsequently, 100 μL of nuclear protein extraction reagent was added to the pellet, followed by incubation on ice for 10 min and centrifugation under the same conditions to extract nuclear protein. The final supernatant containing nuclear protein was aliquoted and stored at −70 °C for later use.

### 2.9 Western blot

A total of 100 mg of liver tissue from each mouse was weighed into a 1.5 mL EP tube, and 900 μL of RIPA lysis buffer containing protease and phosphatase inhibitors was added. The mixture was then homogenized. After lysing on ice for 40 min, the samples were centrifuged at 12,000 rpm for 10 min at 4 °C. The supernatant was collected, and the protein concentration was determined using the BCA method. Subsequently, the protein concentrations were adjusted to the same level, followed by the addition of 5 × loading buffer. The samples were then boiled in boiling water for 10 min. Protein extracts from individual mouse liver samples (*n* = 6 per group) were analyzed separately as independent biological replicates. Subsequent experimental steps, which included sodium dodecyl sulfate-polyacrylamide gel electrophoresis (SDS-PAGE) and exposure, were implemented as previously reported (27).

### 2.10 16s rRNA gene sequencing and analyses

During the last week of drug treatment, mice were individually placed in clean cages lined with sterile filter paper. After defecation, fecal samples were rapidly collected into sterile centrifuge tubes. Once the weight of each sample exceeded 200 mg, the tubes were immediately frozen in liquid nitrogen. The filter paper was then replaced, and feces from the next mouse were collected using the same procedure. Finally, all samples were stored on dry ice and transported to Shanghai Biotree Biological Technology Co., Ltd. (Shanghai, China) for further analysis. The 16S rDNA analysis of fecal samples was performed by the company using established techniques, following the methods previously described (28). Briefly, DNA was extracted and purified from mouse feces using the QIAamp Fast DNA Stool Mini Kit (Qiagen, CA, United States) in strict accordance with the manufacturer’s protocols. Sequencing was performed on the Illumina MiSeq (PE300) targeting the V3-V4 hypervariable regions of the 16S rRNA PCR products, and the data were analyzed in accordance with the aforementioned protocols. Amplified sequences were merged, and operational taxonomic units (OTUs) were delineated using a 97% sequence similarity cutoff. The *α*-diversity and *β*-diversity were analyzed via QIIME2 software, and the dimensionality reduction plots including principal component analysis (PCA), principal coordinate analysis (PCoA), and non-metric multidimensional scaling (NMDS) were generated using R software. Comparative analyses were performed using the Mann–Whitney *U*-test and Kruskal-Wallis test.

### 2.11 Statistical analysis

Statistical analysis and figures were performed using GraphPad Prism 6.0 software (GraphPad Software Inc.). Data were reported as mean ± standard deviation (SD), encompassed a minimum of *n* = 6 biological replicates per experimental group. Student’s *t*-test was used to analyze differences between the two groups, while one-way analysis of variance (ANOVA) was applied for comparisons among multiple groups. In addition, significant effects reported in the variance analysis were also tested using Dunnett’s or Tukey’s *post hoc* multiple comparison tests. Pearson’s correlation analysis was employed to assess correlations. Differences of *p* < 0.05 were considered as statistically significant.

## 3 Results

### 3.1 GPS improved diabetic symptoms and the impaired oxidative stress in T2DM mice

**Figure 1:**
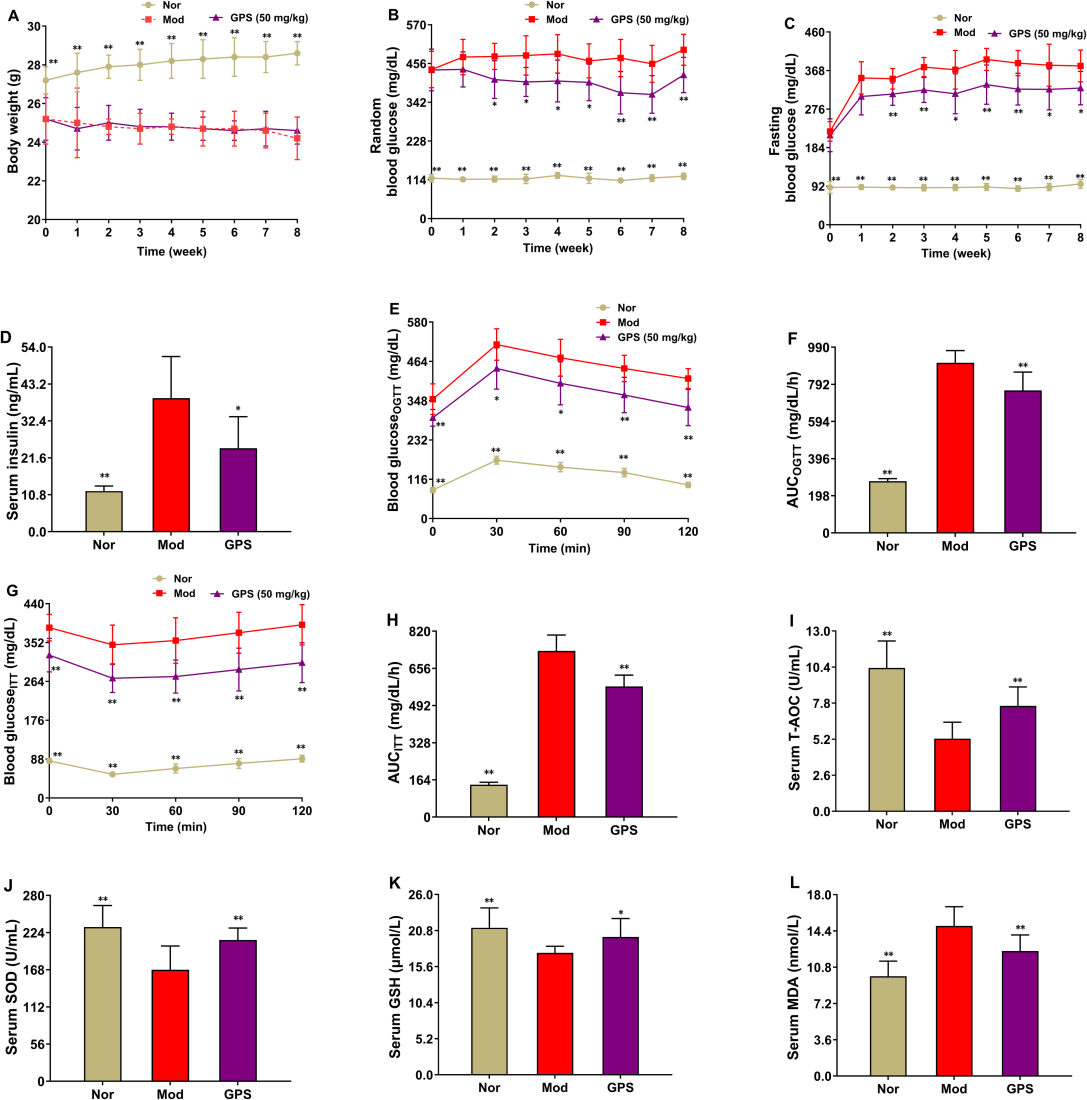
GPS improved diabetic symptoms and the impaired oxidative stress in T2DM mice. **(A)** Body weight. **(B)** Glucose was tested before fasting. **(C)** Glucose was tested after 6 h of fasting. **(D)** Fasting serum insulin levels. **(E)** The blood glucose levels in the oral glucose tolerance test (OGTT). **(F)** Area under the curve (AUC) analysis of OGTT. **(G)** The blood glucose levels in the insulin tolerance test (ITT). **(H)** AUC analysis of ITT. **(I)** T-AOC activities in serum. **(J)** SOD activities in serum. **(K)** GSH levels in serum. **(L)** MDA levels in serum. Data were presented as mean ± SD (*n* = 10). *p < 0.05; **p < 0.01 vs. Mod group.

During the experiment, body weight, FBG, and RBG of the mice were measured weekly to evaluate the therapeutic effect of GPS on T2DM mice. As shown in Figures 1A–D, relative to the Nor group, the levels of RBG, FBG, and insulin in the Mod group were significantly increased, while the body weight in this group was remarkably decreased. In contrast to the Mod group, the levels of RBG and FBG in the GPS group decreased markedly from the second week, and insulin levels were reduced, although GPS exerted no influence on the body weight of T2DM mice. To further evaluate the regulatory effect of GPS on blood glucose levels and islet function in T2DM mice, OGTT and ITT were carried out during the experiment. As illustrated in Figures 1E–H, following glucose loading or insulin injection, blood glucose levels at 30, 60, 90, and 120 min, as well as the AUC values, were remarkably higher in T2DM mice compared to those in the Nor group. On the contrary, GPS treatment dramatically lowered blood glucose levels at these time points and reduced the AUC values relative to the Mod group. To further explore the impact of GPS on OS in T2DM mice, the OS markers present in the serum were measured. As illustrated in Figures 1I–L, compared with the Nor group, the activities of T-AOC, SOD, and the levels of GSH in serum of the Mod group were substantially decreased, while MDA content in serum was markedly increased. Relative to the Mod group, GPS treatment significantly increased the activities of T-AOC, SOD, and levels of GSH in serum, along with a reduction in serum MDA levels. Collectively, these results indicated that GPS could alleviate hyperglycemia and reduce oxidative stress injury in T2DM mice, and may play a central role in hypoglycemic effects.

### 3.2 GPS ameliorated the abnormal pathological morphology of the liver in T2DM mice

**Figure 2:**
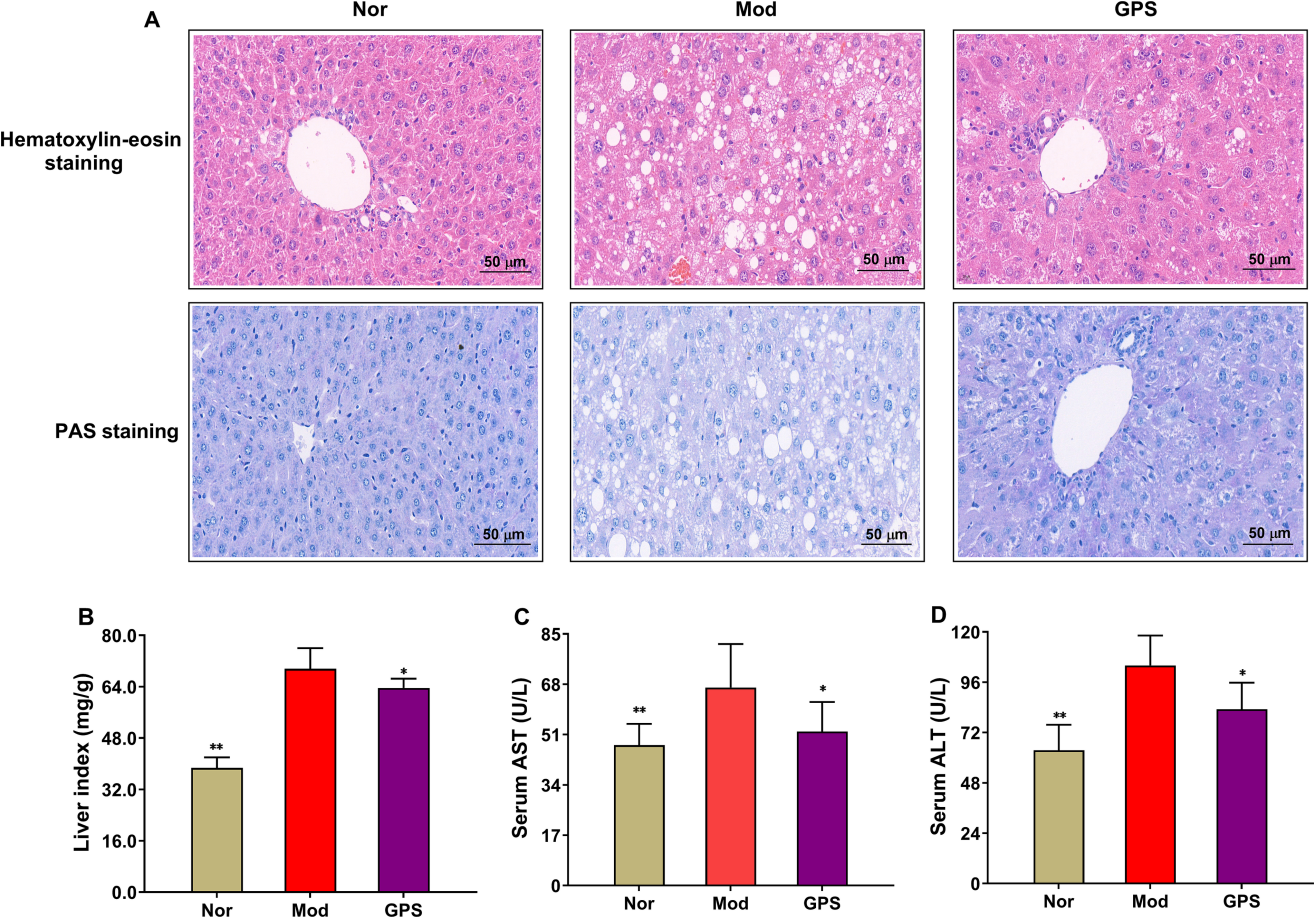
GPS ameliorated the abnormal pathological morphology of the liver in T2DM mice. **(A)** The representative image of HE staining and PAS staining of liver tissue (*n* = 4, photographed at 200×). **(B)** Liver index (*n* = 10). **(C)** AST activities in serum (*n* = 10). **(D)** ALT activities in serum (*n* = 10). Data were presented as mean ± SD. *p < 0.05; **p < 0.01 vs. Mod group.

For the purpose of better comprehending the effect of GPS on liver tissue, HE staining and PAS staining were conducted on the liver tissues obtained from the mice. As shown in Figure 2A, the livers in the Nor group exhibited round and plump hepatocytes, regularly and neatly arranged hepatic plates, no obvious expansion, no protrusion of hepatic sinusoids, and normal adjacent hepatic lobule structures. However, the livers of T2DM mice in the Mod group showed extensive granular degeneration of hepatocytes, loose cytoplasm, some fatty degeneration in a subset of hepatocytes, round cytoplasmic vacuoles of varying sizes, local dilation of hepatic sinusoids, and irregular hepatocyte arrangement. Treatment with GPS partially reversed these pathological abnormalities. Furthermore, after sacrifice, the liver index was calculated, and ALT and AST activities were measured. As illustrated in Figures 2B–D, the Mod group showed significant increases in liver index and serum ALT and AST activities compared to the Nor group. GPS administration markedly reduced these elevated levels relative to the Mod group. All in all, these results indicated that GPS protects liver tissue in T2DM mice from hyperglycemia-induced damage.

### 3.3 GPS alleviated oxidative stress damage of liver in T2DM mice by activating Nrf2/Keap1 signaling pathway

**Figure 3:**
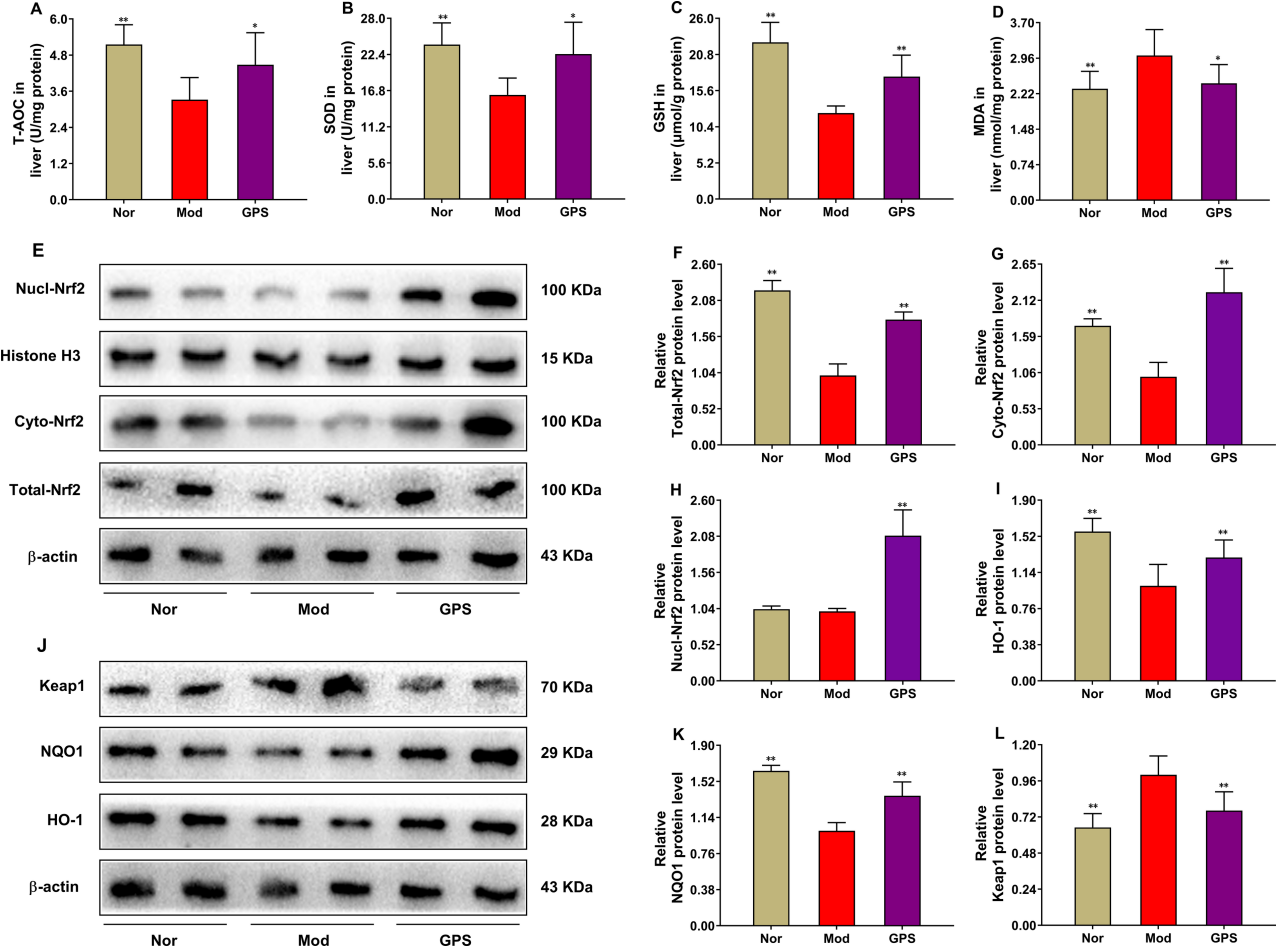
GPS alleviated oxidative stress damage of liver in T2DM mice by activating Nrf2/Keap1 signaling pathway. **(A)** T-AOC activities in liver (*n* = 10). **(B)** SOD activities in liver (*n* = 10). **(C)** GSH levels in liver (*n* = 10). **(D)** MDA levels in liver (*n* = 10). **(E)** Representative western blot images of *β*-actin, total-Nrf2, cytoplasmic Nrf2, histone H3, and nuclear Nrf2 in liver (*n* = 6). **(F–H)** The relative density analysis of total-Nrf2, nuclear Nrf2 and cytoplasmic Nrf2 (*n* = 6). **(I)** The relative density analysis of HO-1 (*n* = 6). **(J)** Representative western blot images of β-actin, HO-1, NQO1, and Keap1 in liver (*n* = 6). (**K, L**) The relative density analysis of NOQ1 and Keap1 (*n* = 6). Data were presented as mean ± SD. *p < 0.05; **p < 0.01 vs. Mod group.

To further explore the molecular mechanism by which GPS improved the abnormal pathological morphology of the liver, OS-related indicators and protein expressions were measured in the livers of T2DM mice. As presented in Figure 3, relative to the Nor group, the activities of T-AOC and SOD, levels of GSH, and protein expressions of Total-Nrf2, HO-1 and NQO1 were noticeably decreased in the Mod group. Meanwhile, MDA levels and Keap1 protein expression were markedly increased. Relative to the Mod group, GPS treatment remarkably increased the activities of T-AOC and SOD, GSH levels, and protein expressions of Nrf2, HO-1, and NQO1, while it obviously reduced MDA levels and Keap1 protein expression. To further investigated the effect of GPS on Nrf2 nuclear translocation, the expression levels of Nrf2 protein in the cytoplasm and nucleus were subsequently determined. The results showed that compared with the Nor group, the cytoplasmic Nrf2 (cyto-Nrf2) protein level in the Mod group was significantly decreased, while there was no significant change in the nuclear Nrf2 (nucl-Nrf2) protein expression. However, after GPS treatment, both the cyto-Nrf2 and nucl-Nrf2 protein expressions were remarkably increased compared with those in the Mod group. Collectively, these results suggested that the hepatoprotective effects of GPS may be related to the activation of the Nrf2/Keap1 signaling pathway, thereby alleviating oxidative stress injury.

### 3.4 GPS attenuated the diversity of GM in the feces of the T2DM mice

**Figure 4:**
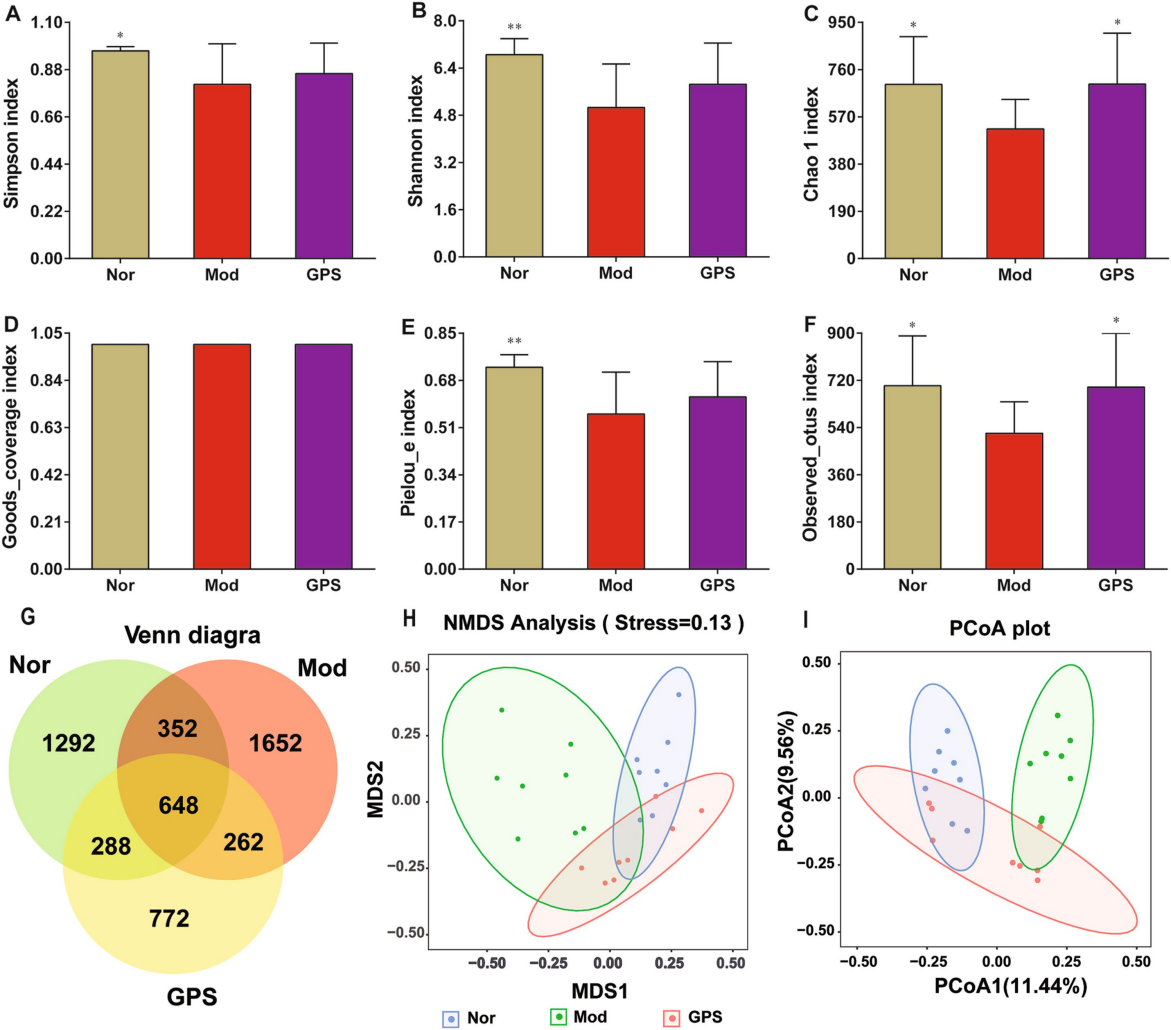
GPS attenuated the diversity of GM in the feces of the T2DM mice. **(A)** Simpson index. **(B)** Shannon index. **(C)** Chao1 index. **(D)** Goods_coverage index. **(E)** Pielou-e index. **(F)** Observed_otus index. **(G)** Venn diagram of OTUs in fecal samples. **(H)** NMDS analysis diagram. **(I)** Principal coordinates analysis (PCoA) of gut microbial communities. Data were presented as mean ± SD (*n* = 6). *p < 0.05; **p < 0.01 vs. Mod group.

To explore the impact of GPS on the GM in the feces of T2DM mice, the composition of the GM was analyzed using 16S rRNA high-throughput sequencing technology. As demonstrated in Figure 4, the Simpson index, Shannon index, Chao1 index, Pielou’s evenness (Pielou-e) index, and Observed_otus index in the Mod group were higher than those in the Nor group, while no significant difference was observed in the Goods_coverage index. After 8 weeks of GPS treatment, compared to the Mod group, the Chao1 and Observed_otus indices in the GPS group increased significantly, and other *α*-diversity indices showed a trend toward normalization. The Venn diagram showed that the Mod group had a higher number of operational taxonomic units (OTUs) than the Nor group, with 2,584, 2,914, and 1,970 OTUs detected in the Nor, Mod, and GPS groups, respectively; 648 OTUs were shared across all groups. At the *β* diversity level, principal coordinate analysis (PCoA) and non-metric multidimensional scaling (NMDS) showed a clear separation between the Nor and Mod groups. After 8 weeks of GPS treatment, the GM in T2DM mice feces exhibited partial restoration, trending toward the composition observed in the Nor group. Overall, these results indicated that GPS reshaped the GM in T2DM mice, suggesting its potential regulatory effect on GM in diabetes.

### 3.5 GPS altered GM composition at the phylum level in T2DM mice

**Figure 5:**
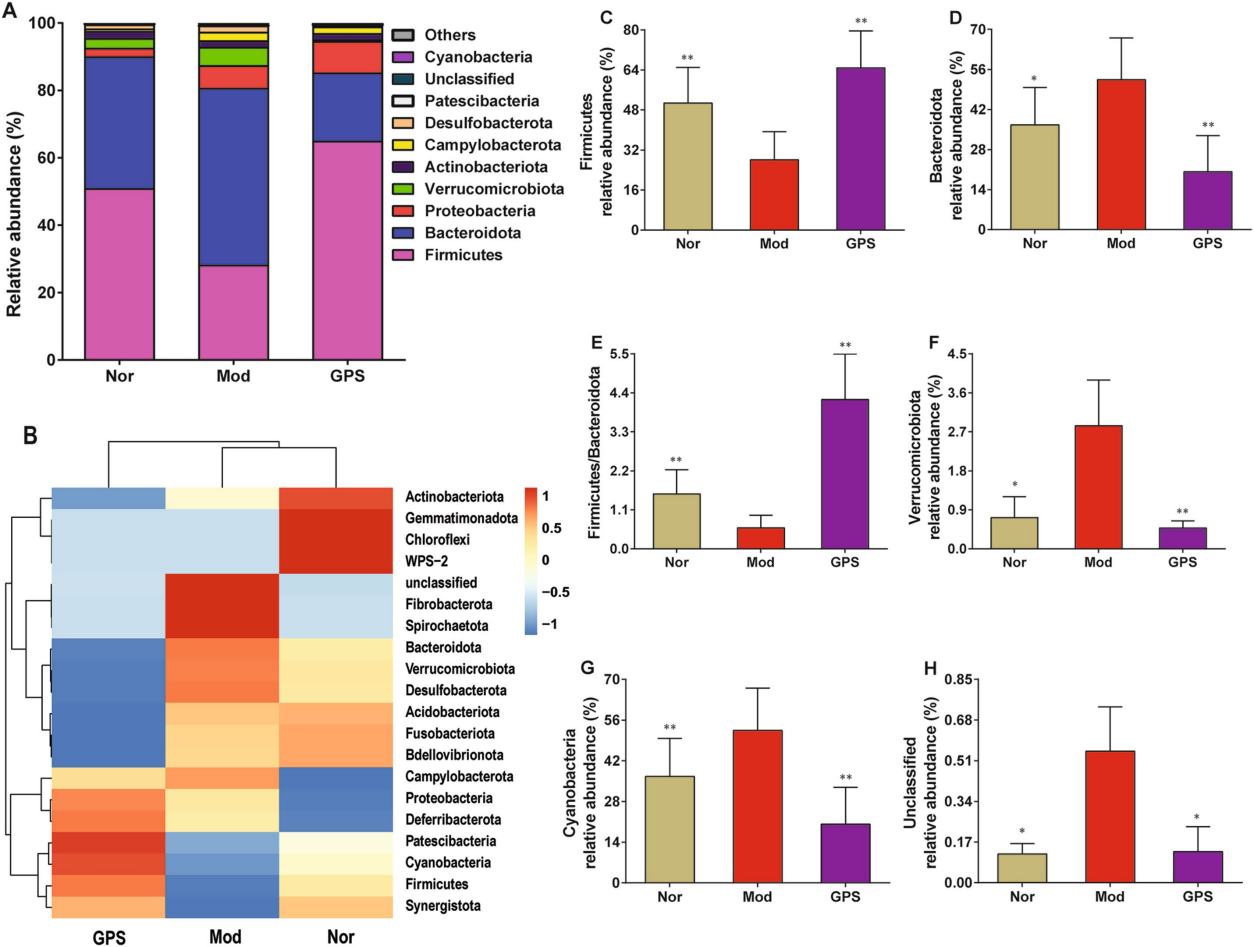
GPS altered GM composition at the phylum level in T2DM mice. **(A)** Relative abundances of species at the phylum level. **(B)** Heatmap analysis of microbial community at the phylum level. **(C)** The relative abundance of *Firmicutes*. **(D)** The relative abundance of *Bacteroidota*. **(E)** The ratio of *Firmicutes* to *Bacteroidota*. **(F)** The relative abundance of *Verrucomicrobiota*. **(G)** The relative abundance of *Cyanobacteria*. **(H)** The relative abundance of *Unclassified*. Data were presented as mean ± SD (*n* = 6). *p < 0.05; **p < 0.01 vs. M group.

The impact of GPS on the composition of the GM was measured at the phylum level. As displayed in Figure 5, the GM in mouse feces mainly consisted of 10 dominant bacterial groups, including Firmicutes, Bacteroidota, Proteobacteria, Verrucomicrobiota, Actinobacteriota, Campylobacterota, Desulfobacterota, Patescibacteria, Unclassified and Cyanobacteria. Compared to the Nor group, the relative abundance of Firmicutes and the Firmicutes/Bacteroidota (F/B) ratio were significantly decreased in the Mod group, while the relative abundance of Bacteroidota, Verrucomicrobiota, Cyanobacteria and Unclassified were markedly increased. After 8 weeks of GPS treatment, these abnormal changes in the GM composition of T2DM mice were significantly reversed. Collectively, these results indicated that GPS plays an important role in maintaining GM homeostasis at the phylum level in T2DM mice.

### 3.6 GPS altered GM composition at the genus level in T2DM mice

**Figure 6:**
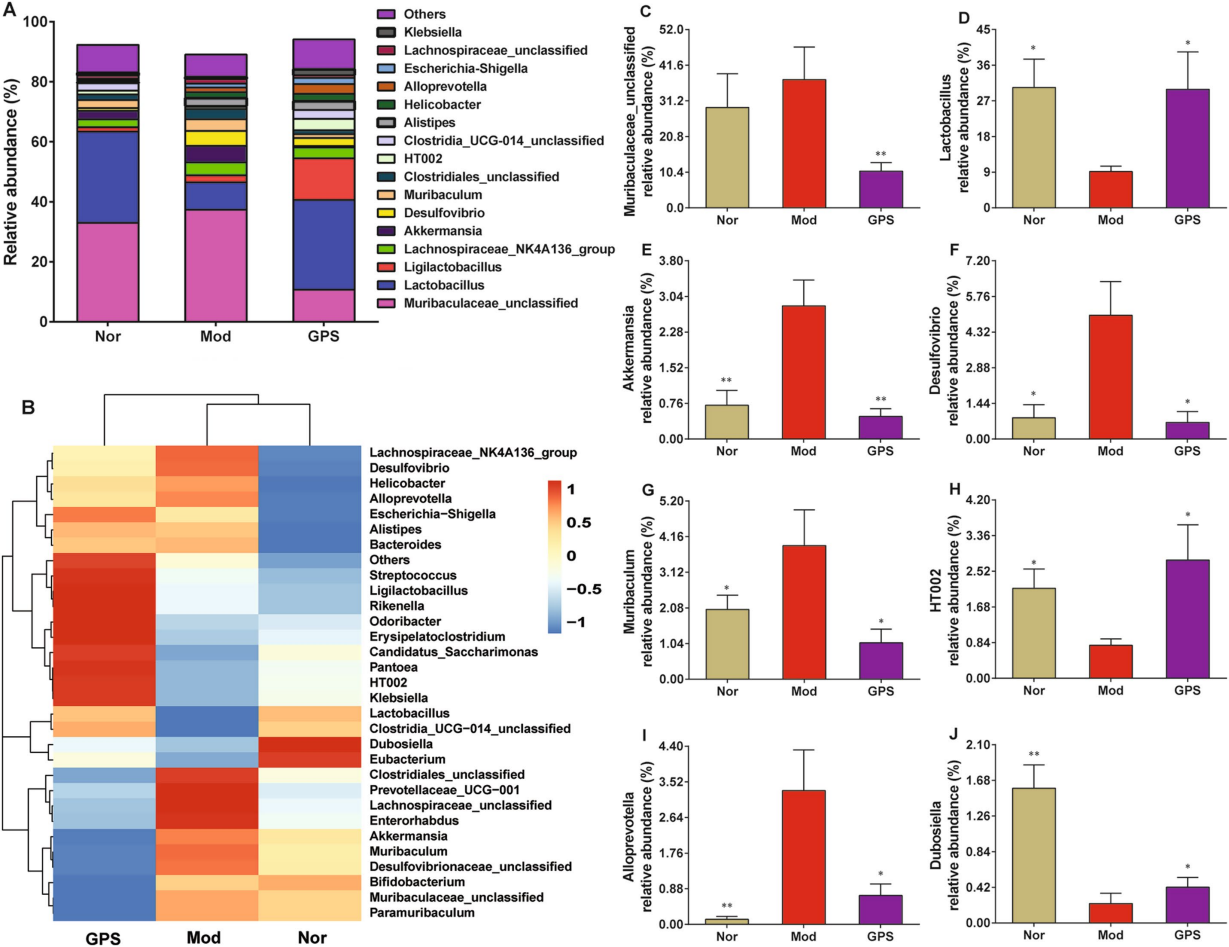
GPS altered GM composition at the genus level in T2DM mice. **(A)** Relative abundances of species at the genus level. **(B)** Heatmap analysis of microbial community at the genus level. **(C)** The relative abundance of *Muribaculaceae_unclassified*. **(D)** The relative abundance of *Lactobacillus*. **(E)** The relative abundance of *Akkermansia*. **(F)** The relative abundance of *Desulfovibrio*. **(G)** The relative abundance of *Muribaculum*. **(H)** The relative abundance of HT002. **(I)** The relative abundance of *Alloprevotella*. **(J)** The relative abundance of *Dubosiella*. Data were presented as mean ± SD (*n* = 6). *p < 0.05; **p < 0.01 vs. Mod group.

To further ascertain the influence of GPS on the GM composition in the feces of T2DM mice, the microbiota was analyzed at the genus level. The results displayed in Figure 6, the fecal GM was mainly composed of 16 dominant bacterial genera, including Muribaculaceae_unclassified, Lactobacillus, Akkermansia, Ligilactobacillus, Desulfovibrio, Lachnospiraceae_NK4A136_group, HT002, Alistipes, Helicobacter, Muribaculum, Clostridiales_unclassified, Clostridia_UCG-014_unclassified, Alloprevotella, Escherichia-Shigella, Lachnospiraceae_unclassified and Klebsiella. Relative to the Nor group, the relative abundance of Muribaculaceae_unclassified, Akkermansia, Desulfovibrio, Muribaculum and Alloprevotella were significantly increased in the Mod group, whereas the relative abundance of Lactobacillus, HT002 and Dubosiella were noticeably decreased. After 8 weeks of GPS supplementation, the abnormal changes in GM composition at the genus level were dramatically reversed. Collectively, these data preliminarily indicated that GPS could improve the composition of GM in feces at the genus level.

### 3.7 Comparison of GM by linear discriminant analysis effect size (LEfSe) analysis and correlation between GM and biochemical parameters

**Figure 7:**
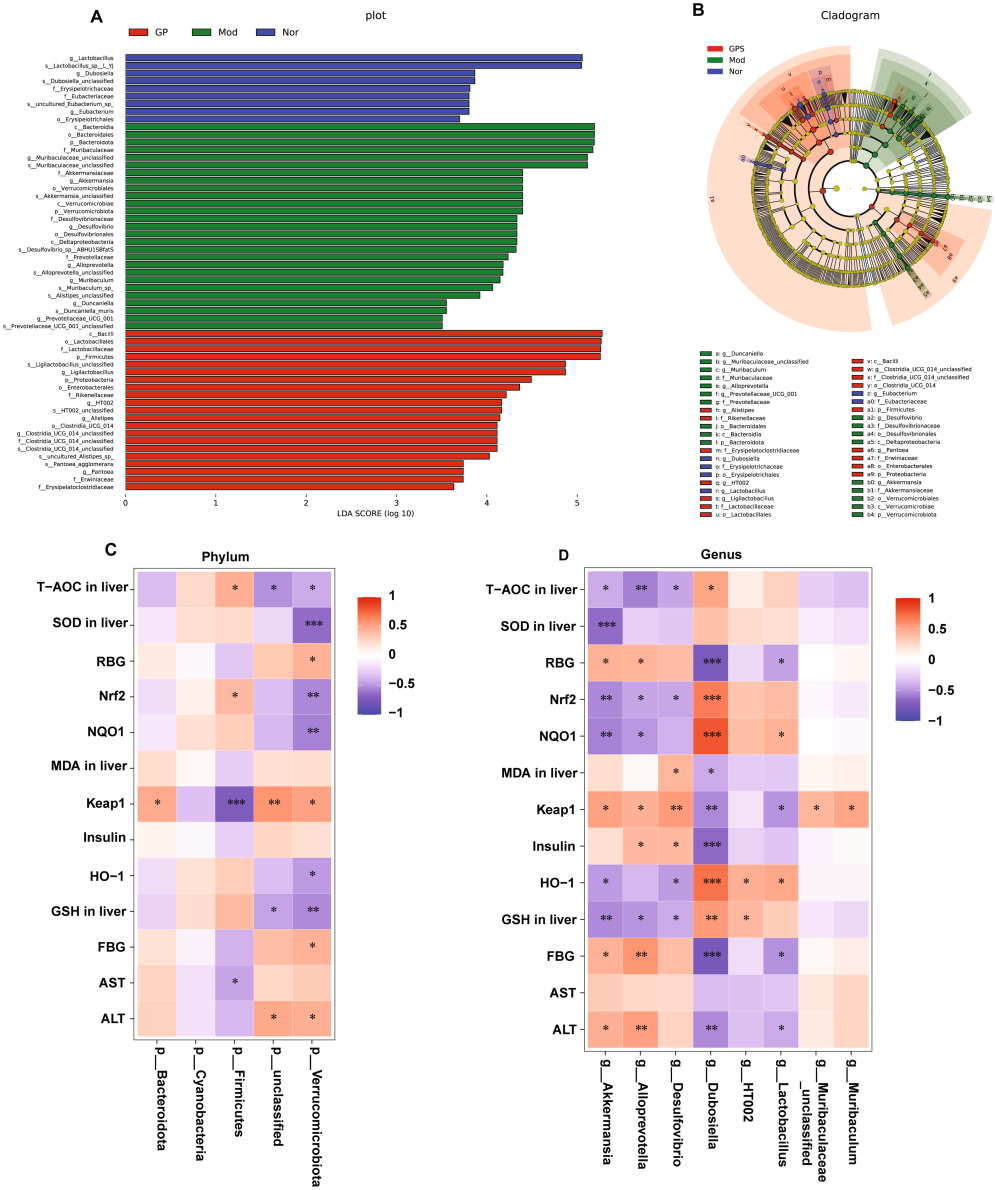
Comparison of GM by linear discriminant analysis effect size (LEfSe) analysis. **(A)** Histogram of LDA values (LDA > 3.5). **(B)** LEfSe cladogram showing differentially abundant bacterial taxa. **(C)** Spearman correlation analysis between intestinal flora and diarrhea-related index at the phylum level. **(D)** Spearman correlation analysis between intestinal flora and diarrhea-related index at the genus level. Data were presented as mean ± SD (*n* = 6). *p < 0.05; **p < 0.01 vs. Mod group.

In addition, LEfSe analysis was performed to identify key bacterial taxa from the phylum to genus levels and to evaluate how bacterial abundance contributed to differences between groups. As demonstrated in Figures 7A,B, using a linear discriminant analysis score threshold of >3.5, a total of 57 significantly different OTUs were identified across the three groups. The number of OTUs in the GPS group tended to approach that of the Nor group. Specifically, 9 biomarkers were found in the Nor group, mainly including genus levels (Lactobacillus, Dubosiella, Eubacterium), species levels (Lactobacillus-sp-L-YJ, Dubosiella-unclassified, uncultured-Eubacterium-sp) and several other genera; 27 biomarkers were identified in the Mod group, mainly including class levels (Bacteroidia, Verrucomicrobiae, Deltaproteobacteria), order levels (Bacteroidales, Desulfovibrionales, Verrucomicrobiales), species levels (Akkermansia-unclassified, Muribaculaceae-unclassified, Desulfovibrio-sp-ABHU1SBfatS, Alloprevotella-unclassified, Muribaculum-sp-, Alistipes-unclassified, Duncaniella-muris, Prevotellaceae-UCG-001-unclassified), family levels (Muribaculaceae, Akkermansiaceae, Desulfovibrionaceae, Prevotellaceae) and several other genera; and 21 biomarkers were present in the GPS group, primarily at family levels (Lactobacillaceae, Rikenellaceae, Clostridia-UCG-014-unclassified, Erwiniaceae, Erysipelatoclostridiaceae), genus levels (Ligilactobacillus, HT002, Alistipes, Clostridia-UCG-014-unclassified, Pantoea), species levels (Ligilactobacillus-unclassified, HT002-unclassified, Clostridia-UCG-014-unclassified, uncultured-Alistipes-sp., Pantoea-agglomerans) and several other genera. To further investigate whether the improvement of diabetic symptoms in T2DM mice by GPS is related to the GM, Spearman correlation analysis was performed between differential microbiota and biochemical indicators at the phylum and genus levels. As indicated in Figures 7C,D, at the phylum level, the abundance of Bacteroidota was positively correlated with the protein Keap1. The abundance of Firmicutes was markedly and positively correlated with T-AOC in liver, the protein Nrf2, along with markedly and negatively associated with the protein Keap1 and the serum AST activities. The abundance of unclassified bacteria showed a strong positive correlation with both Keap1 protein expression and serum ALT activity, while being significantly negatively associated with T-AOC and GSH levels in the liver. The abundance of Verrucomicrobiota was strongly and positively correlated with the RBG, the protein Keap1, FBG and the serum ALT activities, along with dramatically and negatively associated with the T-AOC, SOD and GSH in liver, the protein Nrf2, NQO1, HO-1. At the genus level, the abundance of Akkermansia was strongly positively correlated with random RBG, FBG, Keap1 protein, and serum ALT activity. Conversely, it was significantly negatively associated with liver levels of T-AOC, SOD, GSH, and the protein expressions of Nrf2, NQO1, and HO-1. The abundance of Alloprevotella was strongly and positively correlated with the RBG, FBG, insulin, the protein Keap1and the serum ALT activities, along with remarkably and negatively associated with the levels of T-AOC and GSH in liver, the protein Nrf2 and NQO1. The abundance of Desulfovibrio was strongly and positively correlated with the insulin, the protein Keap1and the MDA levels in liver, along with markedly and negatively associated with the levels of T-AOC and GSH in liver, the protein Nrf2 and HO-1. The abundance of Dubosiella was strongly and positively correlated with the protein Nrf2, NQO1, HO-1, the levels of T-AOC and GSH in liver, along with markedly and negatively associated with RBG, FBG, insulin, the MDA levels in liver, the protein Keap1, the serum ALT activities. The abundance of HT002 was strongly and positively correlated with the protein HO-1and the GSH levels in liver. The abundance of Lactobacillus was strongly and positively correlated with the protein NQO1, HO-1, along with markedly and negatively associated with RBG, FBG, the protein Keap1, the serum ALT activities. The abundance of Muribaculaceae_unclassified and Muribaculum were strongly and positively correlated with the protein Keap1. These results suggested that the improvement of diabetic symptoms in T2DM mice by GPS may be associated with the regulation of GM.

### 3.8 Predictive results of bacterial phenotype

**Figure 8:**
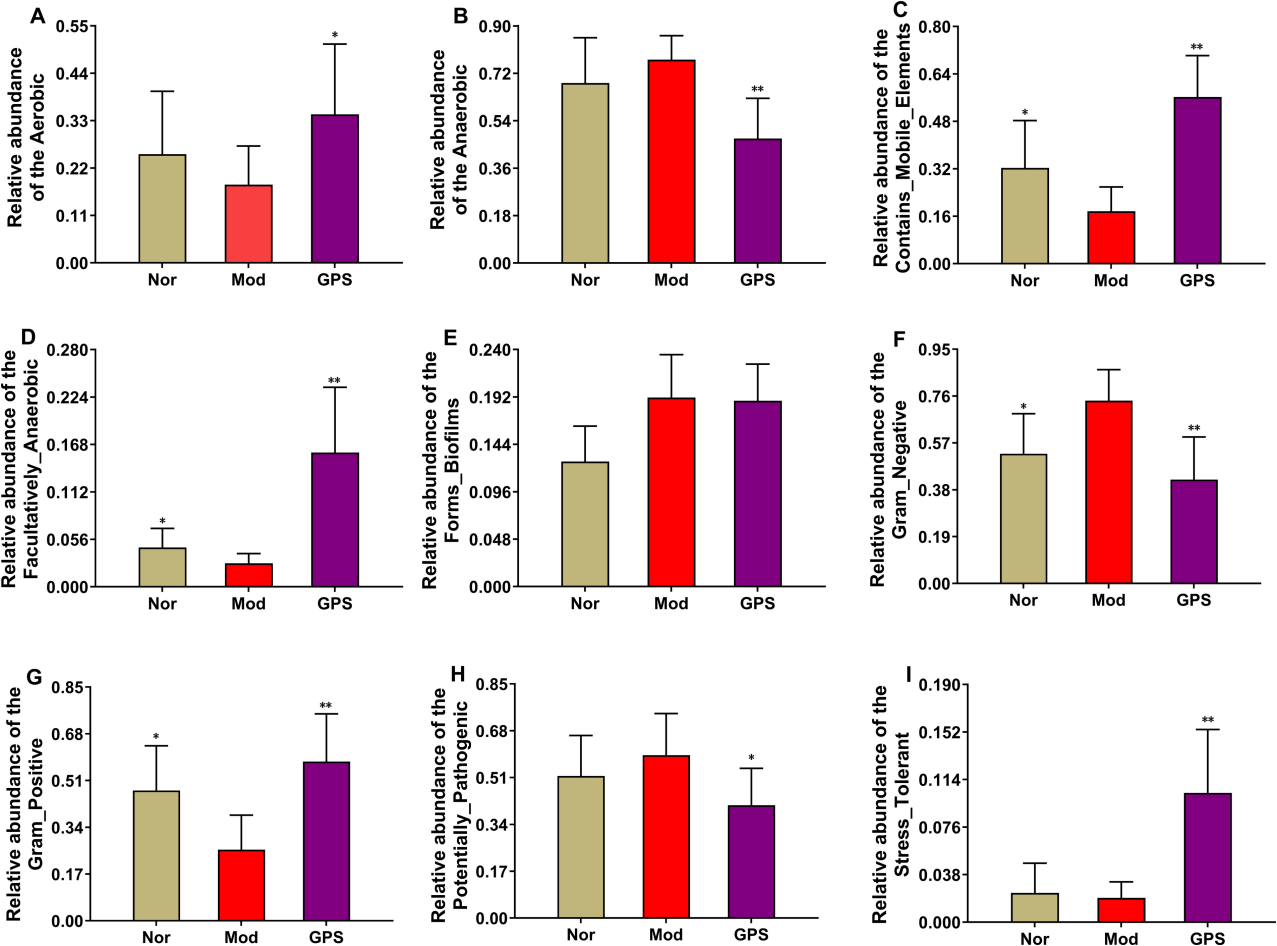
Predictive results of bacterial phenotype. The relative abundance of **(A)** Aerobic, **(B)** Anaerobic, **(C)** Contains mobile elements, **(D)** Facultatively anaerobic, **(E)** Forms biofilms, **(F)** Gram negative, **(G)** Gram positive, **(H)** Potentially pathogenic, and **(I)** Stress tolerant. Data were presented as mean ± SD (*n* = 6). *p < 0.05; **p < 0.01 vs. MOD group.

To better evaluate the effect of GPS on the GM of T2DM mice, BugBase analysis was performed to assess its impact on nine microbial communities closely related to clinical diseases. As indicated in Figure 8, relative to the Nor group, the relative abundance of Contains_Mobile_Elements bacteria, Facultatively_Anaerobic bacteria and Gram-positive bacteria in the Mod group was strongly decreased, while the relative abundance of Gram-negative bacteria increased rapidly, and other microbial communities showed varying trends. Relative to the Mod group, the GPS group showed a significant increase in the relative abundance of Aerobic bacteria, Contains_Mobile_Elements bacteria, Facultatively_Anaerobic bacteria, Gram_Positive bacteria, Stress_Tolerant bacteria, and a noticeable decrease in Anaerobic bacteria, Gram_Negative bacteria and Potentially_Pathogenic bacteria. No statistically significant differences were observed in the other microbial communities.

## 4 Discussion

T2DM is a chronic metabolic disease influenced by genetic factors, poor diet, sedentary lifestyle, and environmental factors. Its global incidence is rising, severely impacting people’s quality of life. Therefore, there is an urgent need to develop safe and effective treatments. The natural compound GPS, extracted from the Chinese herb Gentiana scabra, has gained increasing scientific recognition for its ability to improve metabolic disorders (12, 13). Growing evidence shows that GPS effectively alleviates diabetes and its chronic complications through multiple mechanisms (14–17). Previous studies have mainly focused on how GPS improves T2DM by reducing oxidative stress, inflammatory responses, and gluconeogenesis. However, whether GPS’s anti-T2DM effects involve modulation of the GM remains unclear. Therefore, this study systematically investigated the effects of GPS on liver oxidative stress and the GM in feces, using a T2DM mouse model induced by a HFD combined with STZ injection in C57BL/6J mice. Additionally, correlations between the GM and relevant biochemical indicators were analyzed, thus providing vital experimental support for the further development and application of GPS.

This study successfully replicated the key features of T2DM using a HFD combined with STZ, including weight loss, persistent hyperglycemia, and hyperinsulinemia. Persistent hyperglycemia reduces insulin sensitivity in the liver, adipose tissue, and skeletal muscle, leading to insulin resistance. As a result, pancreatic *β*-cells continuously secrete insulin, causing overwork and structural changes in pancreatic tissue (29). Additionally, chronic hyperglycemia disrupts lipid metabolism, causing lipid metabolic disorders that further induce insulin resistance and oxidative stress. This process inhibits glycolysis, causes blood glucose levels to rise, and exacerbates the progression of T2DM (30). In this study, 8 weeks of GPS dietary treatment dramatically reduced RBG, FBG, and insulin levels. The OGTT is a key diagnostic tool for T2DM. When insulin resistance occurs, glucose utilization by tissues decreases, and glycogen synthesis declines. As a result, blood glucose levels rise significantly after glucose administration and remain above 7.8 mmol/L for an extended period (31). The ITT offers direct, dynamic evidence for the diagnosis of metabolic diseases and evaluating treatment efficacy by measuring insulin-mediated blood glucose regulation (32). In this experiment, blood glucose values and AUC after glucose loading or insulin loading were significantly higher in the Mod group compared to the Nor group, while GPS treatment significantly lowered these values. These findings suggest that GPS has a strong anti-hyperglycemic effect and may serve as a promising candidate for T2DM therapy.

Oxidative damage caused by free radicals is both a cause and a consequence of the development of T2DM and its complications (33). In mice induced by a HFD combined with STZ, prolonged hyperglycemia leads to excessive production of oxygen and nitrogen free radicals, surpassing the body’s ability to neutralize them and resulting in oxidative stress. Increasing evidence shows that high levels of ROS in T2DM impair glucose metabolism in the liver, adipose tissue, and skeletal muscle, disrupt pancreatic structure, and accelerate disease progression (34). Conversely, antioxidant treatment can effectively improve T2DM symptoms and delay disease progression and its complications (35, 36). SOD is one of the core enzymes in the antioxidant defense system, responsible for scavenging superoxide anion radicals, protecting cells from damage, and maintaining oxidative balance (37). T-AOC measures the overall function of the antioxidant system, reflecting the body’s ability to defend against oxidative damage and assess oxidative stress levels (38). GSH is a crucial non-enzymatic antioxidant that maintains cellular redox balance, eliminates harmful substances, and defends against oxidative injury (37). MDA, a primary end product of lipid peroxidation, serves as a direct biomarker of oxidative stress intensity, indicating the extent of cellular damage (37). As expected, T-AOC, SOD, and GSH levels were statistically decreased in the Mod group mice, while MDA levels were markedly increased. In contrast, GPS treatment enhanced antioxidant capacity and reduced MDA content in serum.

The liver is a central metabolic organ that plays a key role in glucose metabolism. In patients with T2DM, excessive hepatic gluconeogenesis increases endogenous glucose production, leading to elevated fasting blood glucose levels (39). Meanwhile, insulin resistance reduces the liver’s sensitivity to insulin-mediated glucose uptake and glycogen synthesis, impairing the ability to store postprandial glucose as glycogen, which further worsens hyperglycemia (40). Furthermore, the liver influences T2DM progression through its roles in lipid metabolism, oxidative stress, inflammation, energy homeostasis, and hormonal signaling networks. Prolonged hyperglycemia generates excessive ROS that damage hepatocyte membranes, disrupt membrane integrity, induce protein carbonylation and DNA strand breaks, interfere with intracellular metabolic signaling, disturb lipid homeostasis, promote *de novo* fatty acid synthesis, and inhibit fatty acid oxidation. This leads to abnormal triglyceride accumulation in hepatocytes (41). Oxidative stress can also drive the progression of diabetic liver toward inflammation and fibrosis by activating pro-inflammatory signaling pathways (42). In this study, GPS significantly improved hepatic steatosis and pathological abnormalities in T2DM mice. It enhanced antioxidant enzyme activity, increased GSH content, and reduced MDA levels in the liver. Nrf2 functions as a key transcription factor responsible for regulating cellular antioxidant responses. During early-stage diabetes, oxidative stress caused by high glucose and hyperlipidemia promotes the dissociation of Nrf2 from its inhibitory protein Keap1 in the cytoplasm. Nrf2 then translocates to the nucleus, binds antioxidant response elements, and activates the transcription of antioxidant genes to protect the liver (43). In late-stage diabetes, Nrf2 expression decreases, leading to the loss of this protective function (44). Additionally, Activating Nrf2 also helps maintain hepatic metabolic homeostasis by regulating lipid metabolism, inhibiting inflammation and fibrosis, thereby slowing the progression of T2DM (45). Here, after GPS treatment, protein expressions of Nrf2, HO-1, and NQO1 were markedly increased in the liver of T2DM mice, while Keap1 protein expression decreased. These results collectively suggest that GPS may alleviate diabetic symptoms by activating the Nrf2 signaling pathway to reduce OS-induced liver damage in T2DM mice. This study is among the first to systematically link GPS-induced Nrf2/Keap1 pathway activation with gut microbiota (GM) remodeling in T2DM. Previous GPS-related studies focused on single targets/pathways or isolated oxidative stress/inflammation alleviation, lacking exploration of crosstalk between hepatic antioxidant signaling and GM homeostasis. Our integrated perspective advances current understanding by demonstrating that GPS exerts anti-T2DM effects through synergistic regulation of both processes: activating Nrf2/Keap1 to enhance hepatic antioxidant capacity and reshaping GM diversity/composition. Correlation analysis confirms their association with improved glycemic control, filling the gap of ignoring their interplay in prior studies.

The GM, often called the “second genome” of humans, plays a crucial role in nutrient absorption and energy balance. It has become a research focus in human health due to its close association with the occurrence and development of T2DM. In patients with T2DM, lo ng-term hyperglycemia and hyperlipidemia mainly alter the diversity and composition of the GM through several mechanisms: (i) persistent hyperglycemia changes the intestinal metabolic microenvironment, remodels the microbial community structure, promotes sugar-loving bacteria, and inhibits anaerobic bacteria (46); (ii) the chronic low-grade inflammation characteristic of T2DM exerts cytotoxic effects and selective pressure on the microbiota, reducing beneficial commensals while favoring drug-resistant and opportunistic pathogens (47); and (iii) hormonal, metabolic, and signaling pathway abnormalities disrupt host-microbiota interactions, creating a cascade of “host dysfunction–interaction imbalance–dysbiosis” (48). In the present study, GPS supplementation improved both *α*-diversity and *β*-diversity in T2DM mice, suggesting that the alleviation of T2DM symptoms by GPS may relate to enhanced GM diversity. Subsequently, the composition of the microbiota was analyzed at the phylum and genus levels. At the phylum level, Bacteroidetes, Firmicutes, and Proteobacteria typically account for over 90% of the microbial community in T2DM mice. Among these, Firmicutes and Bacteroidetes are the dominant phyla in humans, and their ratio is closely linked to microbiota dysregulation in T2DM, obesity, and insulin resistance (49). The F/B ratio is therefore a critical indicator of GM balance (50). Verrucomicrobiota, considered a “protective microbiota” in T2DM, delays the progression of T2DM by enhancing intestinal barrier function and regulating bile acid metabolism (51). Interestingly, GPS treatment markedly increased the F/B ratio but reduced the relative abundance of Verrucomicrobiota, a finding contrary to some studies. These inconsistencies likely reflect environmental factors, natural microbiota variability among individuals, and lifestyle influences (28, 52). Cyanobacteria, identified as a “risk microbiota,” exacerbate T2DM progression by causing mitochondrial damage and endoplasmic reticulum stress in hepatocytes, thus worsening insulin resistance (53). At the genus level, GPS influenced gut bacteria related to barrier protection, inflammation, and metabolism, with the most notable changes observed in Lactobacillus, HT002, Dubosiella, and Desulfovibrio. These results suggest that reshaping the GM’s composition and structure is a key mechanism for GPS’s anti-T2DM effects. Moreover, to further validate this finding, correlation analyses were performed between the GM and relevant biochemical indicators. At the phylum level, Bacteroidota, Firmicutes, unclassified bacteria, and Verrucomicrobiota showed positive correlations with diabetes symptom indexes (FBG, RBG), liver function, oxidative stress markers (ALT, T-AOC), and Nrf2 signaling pathway proteins (Nrf2, Keap1). Negative correlations were observed between Firmicutes, unclassified bacteria, Verrucomicrobiota and other markers such as AST, T-AOC, SOD, GSH, and Nrf2 pathway proteins (Nrf2, NQO1, HO-1, Keap1). At the genus level, differential microbiota correlated significantly with relevant indicators, supported by LEfSe analysis showing distinct intergroup differences. These findings confirm that GPS alters GM composition in association with improved diabetic symptoms. However, it is important to note that the direct functional validation of causal links between Nrf2/Keap1 pathway activation, GM remodeling, and GPS’s anti-T2DM effects is lacking. While correlation analysis demonstrates robust associations between key microbiota, Nrf2 pathway proteins, and improved metabolic phenotypes. Accordingly, we temper our mechanistic conclusion: GPS likely exerts therapeutic effects via synergistic modulation of Nrf2/Keap1-mediated antioxidant defense and GM homeostasis, rather than establishing definitive causal relationships. Future studies will address this gap by performing fecal microbiota transplantation to verify GM’s independent role and using Nrf2-knockout mice to confirm pathway dependence. This limitation does not undermine the study’s value in identifying a novel regulatory axis but highlights the need for follow-up experiments to strengthen mechanistic claims. The amelioration of hepatic steatosis and systemic metabolic dysfunction by GPS may also involve the modulation of other critical nutrient-sensing pathways, such as the mechanistic target of rapamycin (mTOR). mTOR complex1 (mTORC1) serves as a master regulator integrating amino acid and energy signals to control cell growth, metabolism, and oxidative stress (49, 50). In T2DM, chronic nutrient excess leads to persistent mTORC1 activation, contributing to insulin resistance and impaired mitochondrial function, thereby exacerbating oxidative stress (54). Given that microbial metabolites, including specific amino acids, can regulate mTOR activity, the GPS-induced restoration of gut microbiota homeostasis could contribute to normalizing mTOR signaling. Conversely, appropriate mTOR signaling is crucial for maintaining intestinal barrier function and immune responses, which in turn shape the GM composition (55, 56). Therefore, the beneficial effects of GPS on oxidative stress and GM dysbiosis may be interconnected through the modulation of the mTOR pathway, representing a promising area for future investigation to fully delineate its anti-diabetic mechanisms. Additionally, BugBase analysis predicted that GPS significantly affected nine potential microbial biocommunities, but results are primarily exploratory and lack explicit links to oxidative stress, Nrf2 signaling, or glucose metabolism. These findings demonstrate that GPS modulates key bacterial phenotypes: increasing the relative abundance of aerobic, stress-tolerant, and Gram-positive bacteria, while reducing anaerobic, Gram-negative, and potentially pathogenic bacteria, which may indirectly contribute to metabolic improvement by alleviating intestinal inflammation and enhancing barrier integrity-key intermediates connecting gut microbiota to systemic oxidative stress and glucose homeostasis. However, direct causal links between these phenotypic changes and the Nrf2/Keap1 pathway or glycemic control remain unvalidated. Thus, we emphasize these BugBase results as preliminary exploratory data, providing a foundation for future studies to dissect their functional relevance to the core mechanisms identified herein.

A notable limitation of this study is the use of only one GPS dosage (50 mg/kg), which prevents establishing a dose–response relationship and identifying the optimal therapeutic concentration. Incorporating these pertinent data would significantly enhance the credibility and significance of the conclusion. Future research should address this by setting multiple dosage gradients to explore dose-dependent effects on T2DM-related parameters, oxidative stress, and gut microbiota. Another limitation of this study is that although it showed parallel improvements in GM composition and OS parameters following GPS treatment, as well as correlations between specific bacterial communities and OS markers, it did not directly measure GM-derived metabolites such as short-chain fatty acids, bile acids, or lipopolysaccharides, which hinders the elucidation of the relationship between GM and OS. Future research will quantify these metabolites to clarify their role in GPS-mediated crosstalk between GM and hepatic OS, which will strengthen the mechanistic link between the two key pathways explored herein.

**Figure 9:**
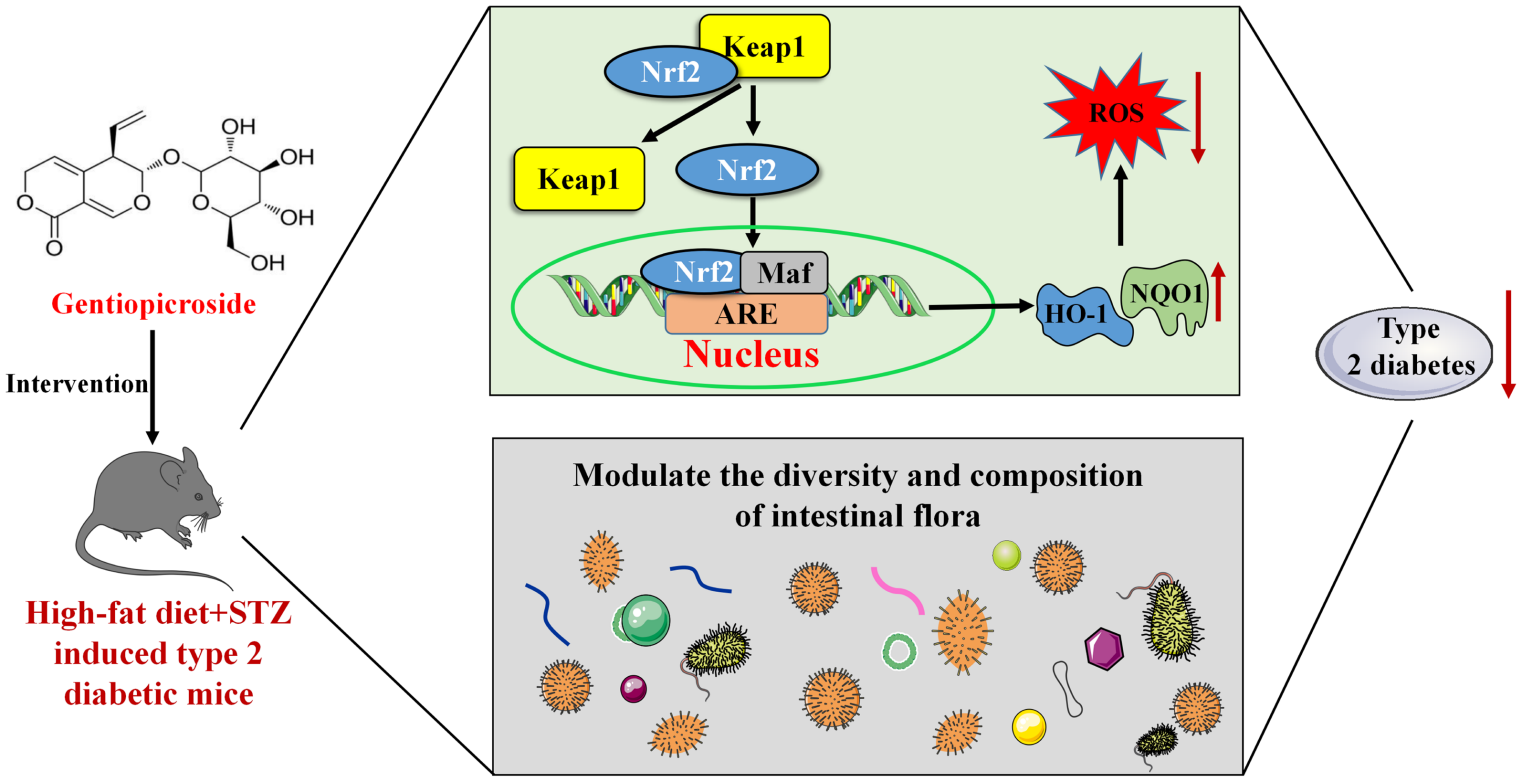
Dietary treatment with GPS improves diabetic symptoms in T2DM mice may by alleviating hepatic oxidative damage through activating the hepatic Nrf2/Keap1 signaling pathway and improving the diversity and composition of the gut microbiota.

In conclusion, GPS supplementation preliminary reduced blood glucose levels, improved glucose tolerance, enhanced insulin sensitivity, and alleviated abnormal liver pathology in T2DM mice. Its therapeutic effects may stem from alleviating hepatic OS by activating the Nrf2/Keap1 signaling pathway and maintaining metabolic homeostasis through improving GM diversity and composition (Figure 9). This study highlights GPS’s potential as a treatment for T2DM and provides important experimental evidence for its further development. Future research should focus on identifying the key GM involved in the anti-T2DM effect of GPS.

## Data Availability

The 16s rRNA sequencing data presented in this study has been deposited and made publicly available in the NCBI repository under accession number PRJNA1427581.
